# Assessment of grazing livestock balance on the Eastern Mongolian Plateau based on remote sensing monitoring and grassland carrying capacity evaluation

**DOI:** 10.1038/s41598-024-84215-4

**Published:** 2024-12-30

**Authors:** Menghan Li, Juanle Wang, Kai Li, Yaping Liu, Altansukh Ochir, Davaadorj Davaasuren

**Affiliations:** 1 State Key Laboratory of Resources and Environmental Information System, Institute of Geographic Sciences and Natural Resources Research, Chinese Academy of Sciences, Beijing, 100101 China; 2 College of Geoscience and Surveying Engineering, China University of Mining & Technology (Beijing), Beijing, 100083 China; 3 Jiangsu Center for Collaborative Innovation in Geographical Information Resource Development and Application, Nanjing, 210023 China; 4 Environmental Engineering Laboratory, Department of Environment and Forest Engineering, School of Engineering and Applied Sciences, Institute for Sustainable Development, National University of Mongolia, Ulaanbaatar, 14201 Mongolia; 5 Department of Geography, School of Art and Sciences, National University of Mongolia, Ulaanbaatar, 210646 Mongolia

**Keywords:** Mongolian Plateau, Grass-livestock balance, Remote sensing, Grassland productivity, Livestock husbandry, Environmental impact, Carbon cycle

## Abstract

Rational utilization of natural resources is crucial in arid and semi-arid areas due to their vulnerable ecosystems and low resource resilience. Achieving a balance between grassland production and livestock grazing, known as the pasture-livestock balance, is essential for the sustainable development of grassland resources on the Mongolian Plateau (MP). This study focuses on the grassland regions of 8 provinces in eastern Mongolia (MNG) and 7 leagues in Inner Mongolia (IMNG), China, during the period from 2018 to 2022. Machine learning methods were employed for land cover classification and above-ground biomass (AGB) estimation. The grassland carrying capacity was assessed using the grassland carrying capacity index (GCC). The results indicate that: (1) The grassland classification accuracy on the MP exceeds 95%, with grassland area accounting for approximately 47% of the total.(2)The AGB of the grasslands exhibits a clear spatial heterogeneity, increasing from southwest to northeast. Additionally, nearly 80% of the grassland productivity is of high quality, reaching up to 250 g/m^2^.(3) Between 2018 and 2022, the MP exhibited a relatively high grassland carrying capacity, with an average of 1.8 SU/ha. However, the overall grassland carrying condition has gradually deteriorated, primarily due to factors such as grassland fires and an increase in livestock numbers. Based on the varying degrees of grassland degradation, different policy recommendations have been proposed. This study approach, findings and policy suggestions are significant for the development of livestock farming and grassland management on the MP.

## Introduction

The grassland ecosystem is one of the most widespread ecosystems in arid and semi-arid regions, closely related to the growth and development of flora and fauna, ecological and environmental safety, and regional socio-economic development. However, over the past few decades, with continuous climate change and societal development, the degradation of grassland ecosystems has become increasingly significant^1–3^. Climate factors, such as global warming and changes in precipitation resulting from the greenhouse effect, significantly impact grassland degradation. Among these factors, anthropogenic influences, especially grazing, interfere more rapidly and directly with grassland degradation. Reasonable grazing activities can promote soil moisture and nutrient cycling in grasslands. However, long-term overgrazing may lead to permanent damage to grassland productivity, ultimately accelerating the process of grassland degradation. Therefore, accurately assessing the balance between grassland productivity and livestock grazing intensity is crucial for the sustainable development of grassland resources^4,5^.

The grazing-livestock balance is critical to the development of pastoral animal husbandry. It involves controlling livestock numbers and grazing intensity within a particular grassland area to avoid overgrazing and overloading, thereby reducing grassland degradation and damage to the grassland ecosystem. This ensures a dynamic equilibrium between grassland resource supply and the production needs of animal husbandry^6–8^. Its core objective is to achieve the sustainable utilization and conservation of grassland ecosystems while ensuring the sustainable development and ecological security of animal husbandry^9,10^. Grazing-livestock balance exhibits multi-level characteristics across different stages. Briefly, it can be divided into three levels: first, achieving a dynamic equilibrium between forage production and livestock consumption; second, ensuring beneficial changes in grassland biodiversity while meeting the foraging needs of livestock; and finally, achieving sustainable and healthy development at the ecosystem level of grasslands^11–13^. Qu et al. analyzed the dynamic balance between grass and livestock in the Xilingol League grasslands of Inner Mongolia (IMNG) from 2000 to 2015 using remote sensing data and field measurements, and it was found that nearly half of the area in the southwestern part is affected by overgrazing^14^. Lu et al. estimated the optimal livestock unit number for Etoke Front Banner, Inner Mongolia, in 2030 based on a water-land-grass balance model, it is expected that the livestock farming scale will reach 1.18 million head^15^. In the study of grass-livestock balance, most scholars focus exclusively on the conditions in Mongolia (MNG) or IMNG, with a notable lack of research addressing the overall grass-livestock situation across the transboundary grassland belt in MP. Meanwhile, this region has more population and high livestock developing pressures. Therefore, there is a notable gap in studies addressing the grazing-livestock balance across the eastern Mongolian Plateau (MP).

The key to evaluating the grazing-livestock balance lies in accurately estimating aboveground grassland biomass and using this estimate to determine grassland carrying capacity^16–18^. The direct measurement method is the most effective approach for estimating grassland production, but it requires significant human and material resources and cannot be applied to large-scale predictions^19,20^. On the other hand, model simulation methods based on remote sensing data possess universality and a certain degree of accuracy^21–24^. For instance, the Normalized Difference Vegetation Index (NDVI) is commonly used to construct models for estimating aboveground grassland biomass, as it can reliably reflect changes in grassland biomass^25–28^. Currently, the commonly used remote sensing data are MODIS and Landsat data. Due to the high temporal resolution of MODIS data and the high spatial resolution of Landsat data, both have been widely used in grassland biomass estimation. Additionally, the Google Earth Engine (GEE) platform not only provides a vast array of data beyond the aforementioned MODIS and Landsat datasets, but also enables online cloud computing for remote sensing data processing, significantly enhancing computational efficiency and offering tremendous potential in remote sensing applications.

Grassland carrying capacity refers to the quantity and intensity of livestock production activities that the grassland ecosystem can sustain and maintain over a certain period, reflecting the adaptability and capacity of grassland resources to accommodate livestock production^29–34^. As one of the world’s most extensive temperate grasslands and currently one of the most well-preserved grasslands, the MP covers an area of nearly 1.5 million square kilometers^35–37^. In recent years, climate and environmental changes caused by global warming, overgrazing by herders, and inadequate ecological restoration after natural resource extraction have had varying degrees of impact on nearly 70% of the MP. Additionally, nearly 10% of the region has experienced desertification^38^. Many grasslands have suffered severe degradation, resulting in decreased productivity, proliferation of poisonous and invasive weeds, and posing a significant threat to the safety of the ecological environment and the supply of economic resources^39–41^.

To clarify the grazing-livestock balance on the eastern MP and further aid in improving its ecological environment., this study aims to: (1) Estimate the aboveground biomass of grasslands on the eastern MP based on remote sensing data and meteorological data, and use this estimate as a basis for a rapid assessment of grassland carrying capacity. (2) Combine statistical data on animal husbandry on the MP to investigate the grazing-livestock balance situation from 2018 to 2022 and provide recommendations to support grassland resource management and the sustainable development of animal husbandry on the eastern MP.

## Result

### Biomass on grassland

**Fig. 1:**
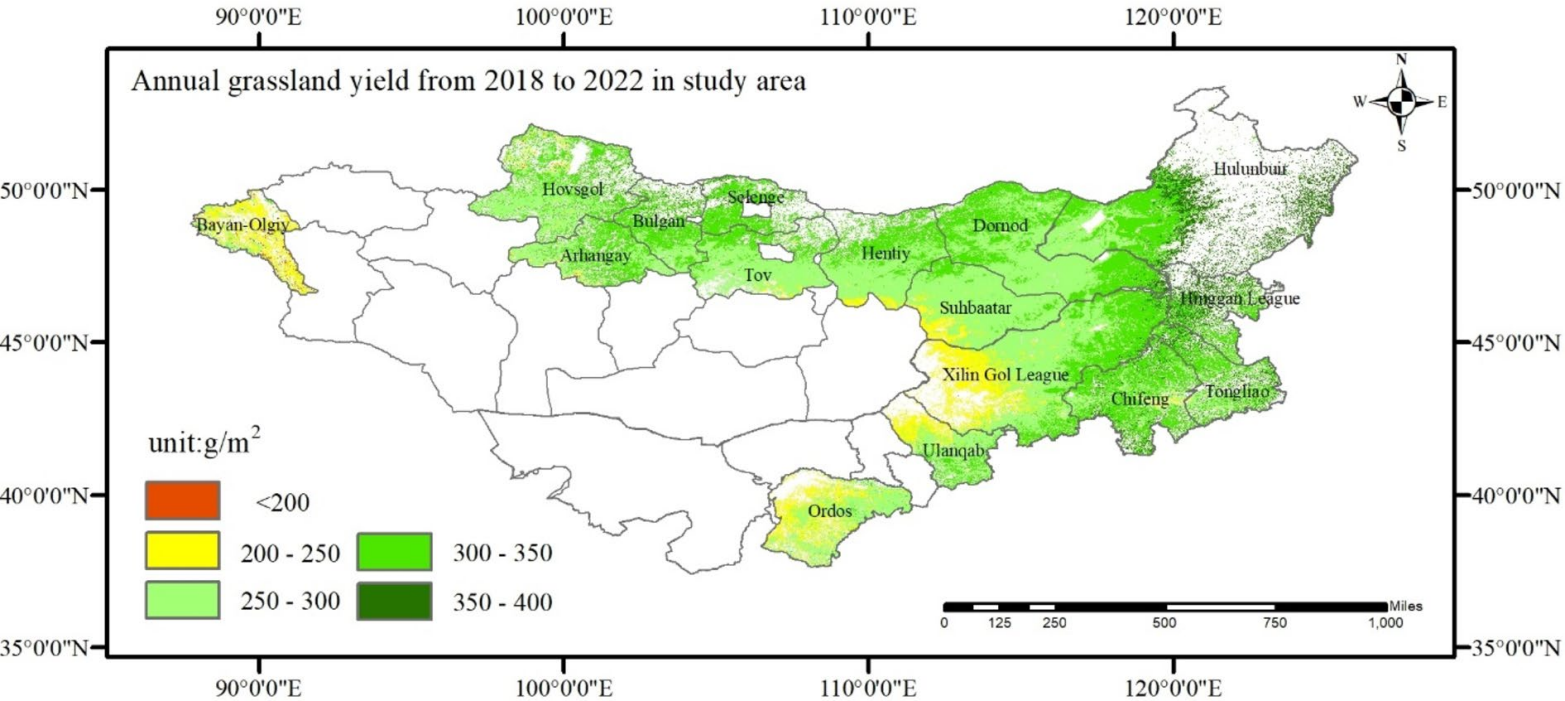
Distribution map of average grassland yield from 2018 to 2022.

The biomass data for grassland on the eastern MP were inverted and validated based on 327 measured grassland biomass sample points, achieving a correlation coefficient of 0.78 and an RMSE of 37.8 g/m^2^. As shown in (Fig. 1). From a spatial perspective, areas with low and extremely low grassland productivity on the MP are concentrated in the western three provinces of MNG (Khovd, Bayan-Ölgii, Uvs), the northern part of East Gobi Province, and the western grasslands of Xilin Gol League in Inner NG. These regions are adjacent to desert and barren areas, characterized by high temperatures and limited water sources. The ecological environment is fragile, making them prone to grassland degradation and resulting in lower grassland productivity. High grassland productivity values are concentrated in Hulunbuir City in IMNG, the Selenge River Basin in MNG, and the eastern three provinces (Dornod, Khentii, Sukhbaatar). These regions benefit from sufficient precipitation and moderate temperatures, fostering diverse and stable ecological systems. They serve as primary grassland growth areas and are central to livestock farming on the MP. Overall, the distribution of grassland productivity on the MP exhibits significant spatial heterogeneity, with grassland biomass gradually increasing from southwest to northeast.

### Livestock population statistics

**Fig. 2:**
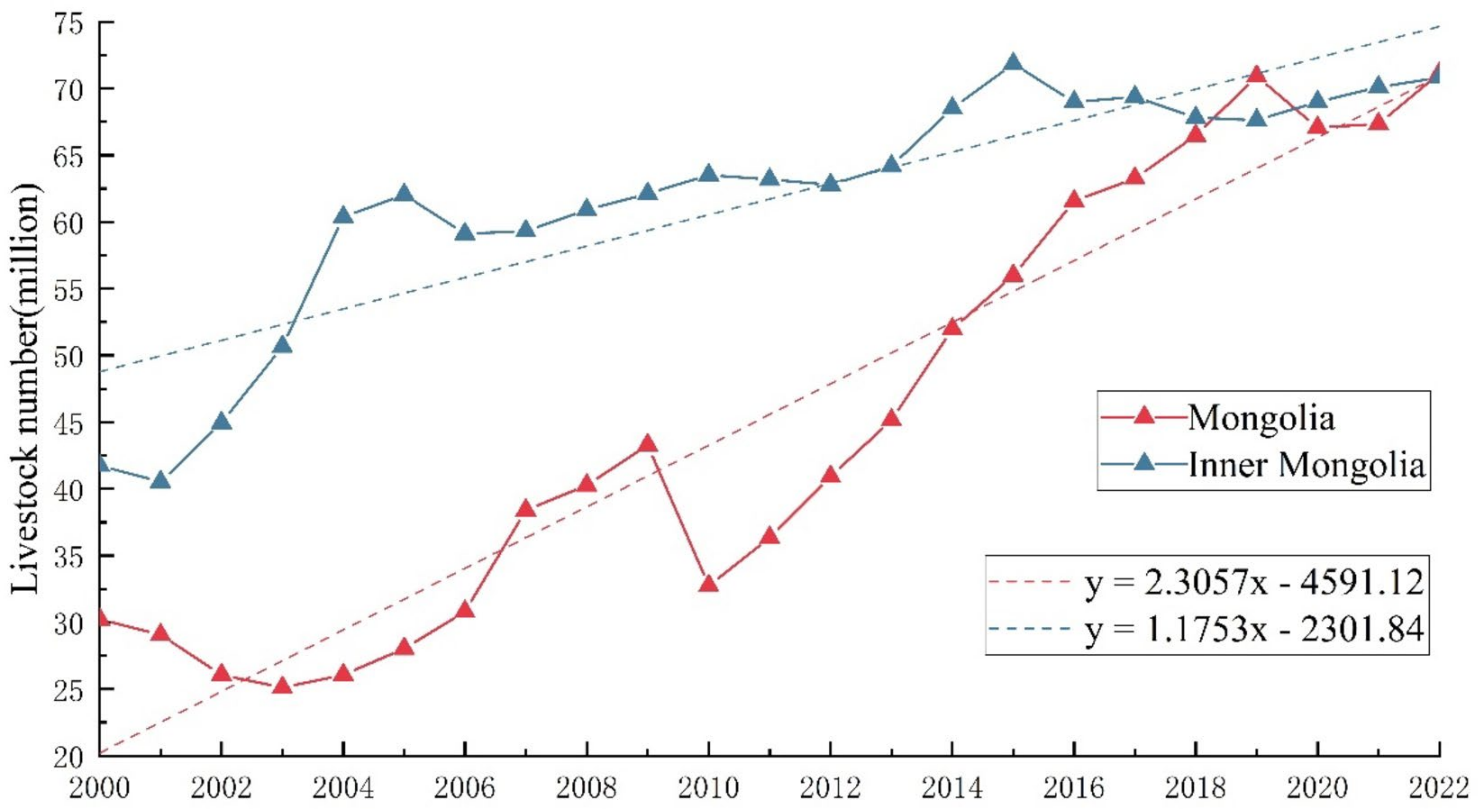
Livestock number in Mongolia and inner Mongolia in recent 23 years.

There is a significant difference in the total livestock population between MNG and IMNG in China, as shown in (Fig. 2). The livestock quantity in IMNG reached nearly 45 million heads as early as 2000, experiencing a significant increase between 2000 and 2005. After this period, the growth of the livestock population gradually stabilized, showing a steady upward trend. In contrast, the livestock population in MNG was only around 30 million heads at the beginning of 2000. Between 2000 and 2010, the total livestock number increased slowly, with a sharp decline occurring in 2010. Extreme weather events were one of the reasons for the decrease in livestock numbers. The severe winter disaster (dzud, meaning harsh winter conditions in Mongolia) during the winter of 2019 resulted in the death of 10 million livestock, which accounted for 23.4% of the total population, significantly impacting the development of the livestock industry in MNG. Between 2010 and 2019, there was a sharp increase in the livestock population in MNG, which gradually stabilized afterward. In terms of overall trends, the growth rate of the livestock population in MNG is nearly twice as fast as that in IMNG, reaching an average annual growth of 2.3057 million heads. In comparison, Inner Mongolia’s average annual growth is 1.1753 million heads.

According to the conversion method mentioned earlier, standard livestock unit (SU) statistics were conducted for the main grassland production areas on the eastern MP, including 11 provinces of MNG and seven prefecture-level leagues (cities) of IMNG. The specific data are shown in (Table 1). The region with the highest actual livestock units on the MP is Chifeng City, where the annual livestock units exceed 16 million standard units. The main reason for this is that the livestock industry in this city is primarily focused on large cattle, with extensive cattle accounting for 46.6% of the converted livestock units, while cows contribute 37.4% of the livestock units. As the second-ranked region in terms of livestock units, Tongliao City’s overall situation is like that of Chifeng City. Bayan-Ölgii Province, located in the western part of MNG and adjacent to the southern desert region, faces significant environmental challenges and has the lowest livestock units in 2022. The province experiences harsh ecological conditions and has a limited grassland area, which hampers the development of its livestock industry. Khövsgöl Province, a significant livestock farming base in MNG, consistently maintains around 11 million standard livestock units throughout the year and has consistently had the highest number of livestock units in Mongolia. From a temporal perspective, the SU in all regions has been increasing, with the average growth rate of SU in each region reaching 17.8%. Among them, the SU growth rate in the eastern provinces reached as high as 49%, while the SU in Chifeng City remained the most stable, increasing by only 1.9%.

**Table 1:** Statistics of the actual standard number of livestock units (SU) in various provinces and cities of MP.

Region	Actual standard livestock units (thousands)				
	2018	2019	2020	2021	2022
Hovsgol	9483.96	10082.73	9433.97	10347.45	11268.4
Bayan-Olgiy	3448.13	3510.11	3540.17	3886.2	3815.6
Dornod	5220.39	5747.55	6165.7	6968.9	7808.64
Bulgan	5948.87	6575.38	6170.74	6698.32	7431.89
Selenge	3159.69	3406.14	3691.41	3952.51	4119.51
Hentiy	8179.14	8573.55	8324.36	8922.71	9361.77
Arhangay	10714.02	12107.65	10977.67	11528.89	12620.81
Tov	7905.73	8624.24	8535.02	8348.5	9097.56
Suhbaatar	6851.17	6998.02	7186.34	7828.16	8652.77
Chifeng	16929.07	16372.3	16452.82	16695.11	17253.57
Ordos	8482.59	9213.55	9384.56	9684.55	9908.21
Hulunbuir	12087.85	12004.12	11766.57	12328.2	12971.67
Tongliao	14779.59	15087.98	15951.88	16610.07	17776.86
Ulanqab	4769.49	4630.91	4889.84	4949.58	5018.42
Xilin Gol League	11506.66	11448.45	11662.44	12157.16	12802.31
Hinggan League	9942.4	10592.59	11275.37	11185.56	11429.695

### Grassland carrying capacity calculation

**Fig. 3:**
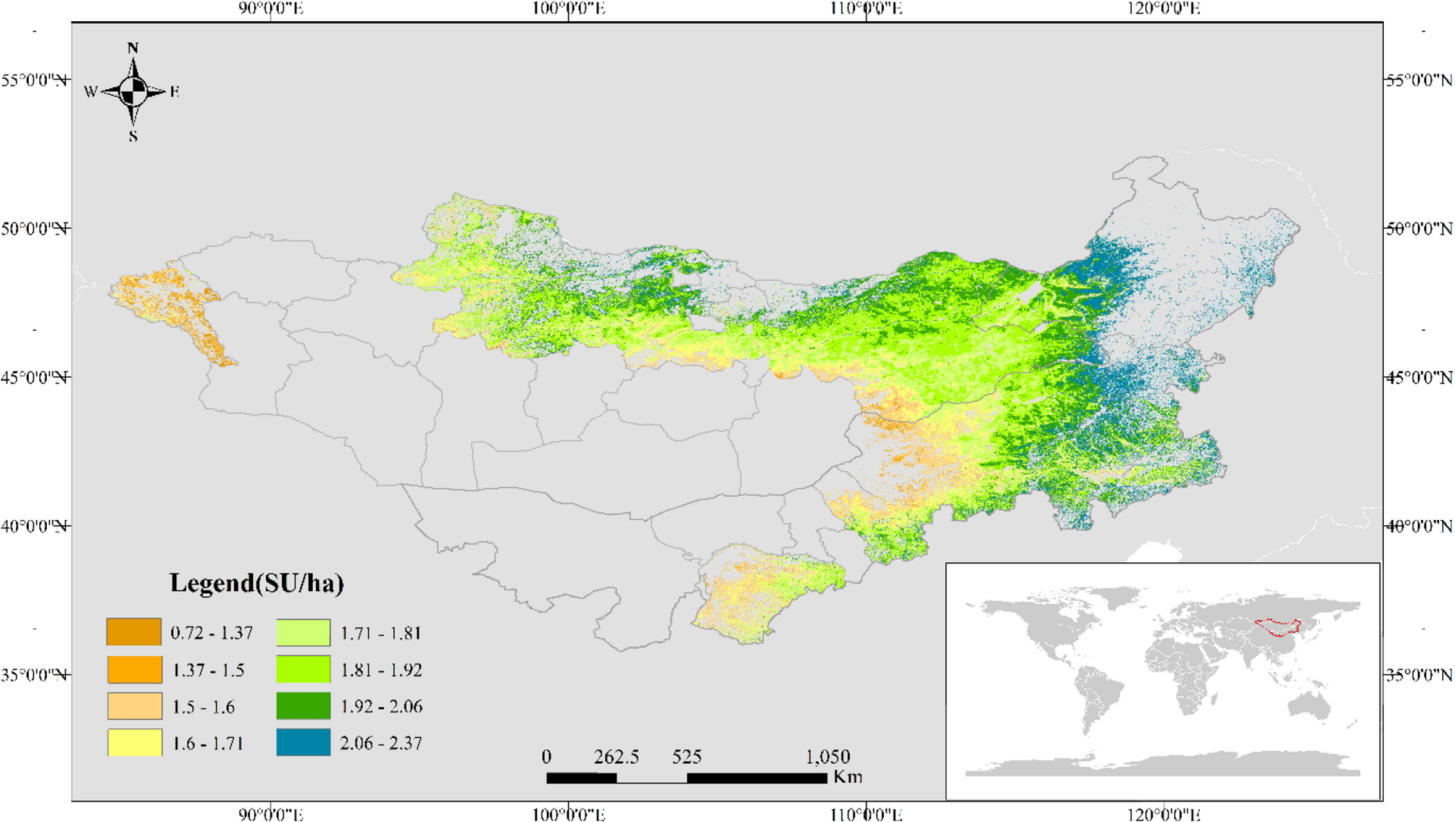
Distribution of grassland carrying capacity on MP.

The carrying capacity of grasslands on the MP exhibits spatial characteristics, with lower values in the western region and higher values in the eastern region, as shown in (Fig. 3). In the western grasslands, the carrying capacity generally remains below 1.6 SU/ha, whereas in the east, it can reach up to 2 SU/ha. Furthermore, there is a gradual increase in carrying capacity from West to East. The overall carrying capacity of grasslands in MNG is significantly lower than that in IMNG. Among the provinces in MNG, Selenge, Dornod, and Khentii are the primary areas for high-quality livestock grazing. In contrast, the overall carrying capacity of grasslands in Inner Mongolia is relatively high, with most grasslands having a carrying capacity of over 1.8 SU/ha. Regions such as Hulunbuir, Xilingol League, Chifeng, and Tongliao possess large areas of high-quality grasslands that meet most of Inner Mongolia’s livestock needs.

### Mongolian Plateau grazing-livestock balance analysis

**Fig. 4:**
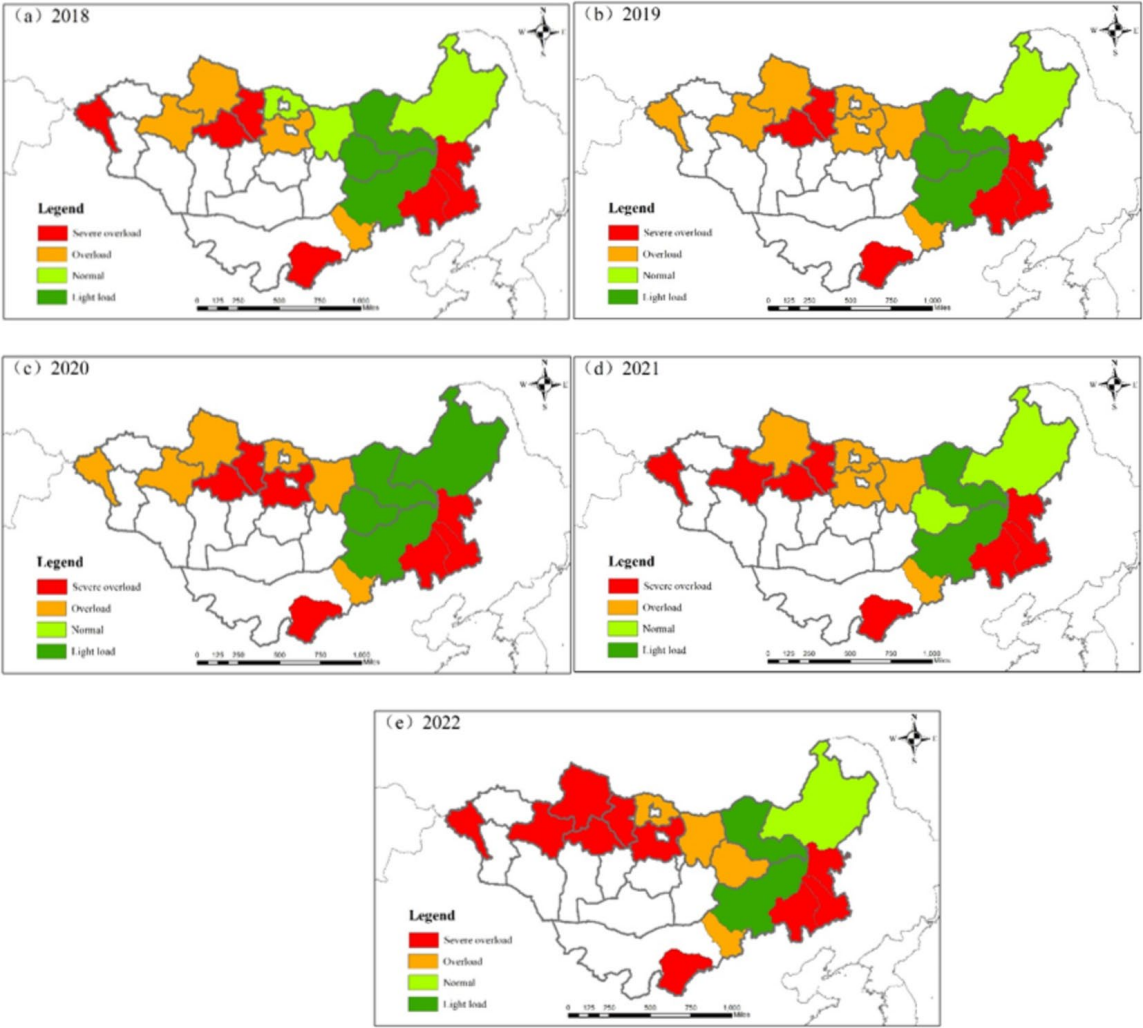
Grassland and livestock balance in the Mongolian Plateau region from 2018 to 2022.

As shown in Fig. 4, the active regions of grass-livestock balance changes on the eastern MP are primarily in MNG. The area west of Bulgan Province in MNG consistently remains between overload and severe overload throughout the year, indicating heavy overgrazing. Moreover, by 2022, the situation in this area had deteriorated to severe overload. In the region east of Bulgan Province, the grassland carrying capacity has significantly declined over the past five years. In 2018, only the Central Province in this area was experiencing overload. By 2022, except for the Eastern Province, all other areas had either overloaded or severely overloaded grasslands. The grassland carrying capacity in various prefecture-level regions of IMNG is relatively stable. However, cities such as Chifeng, Tongliao, Hinggan League, and Ordos have consistently been in severe overload for an extended period. Hulunbuir and Xilingol League, the primary livestock bases in Inner Mongolia, have maintained their grasslands at normal and light carrying capacities for an extended period.

Moreover, Hulunbuir transitioned to light carrying capacity in 2020. The overall grassland ecological environment remains relatively stable, indicating significant potential for further development in the livestock industry. The grassland carrying capacity has deteriorated most rapidly in the provinces of Hovsgol, Hentiy, Zavkhan, and Suhbaatar. Among them, the rapid increase in SU numbers in Hovsgol and Suhbaatar provinces has led to environmental degradation of the grasslands, with both provinces experiencing SU growth rates exceeding 20%. In contrast, the deterioration in Zavkhan and Hentiy provinces is primarily due to a decline in grassland productivity, resulting in the land’s inability to support comparable SU numbers. Overall, the grassland carrying capacity on the eastern MP is continuously deteriorating, with MNG experiencing particularly significant degradation. Although changes in grassland carrying capacity in Inner Mongolia may not be as apparent, the grassland carrying state index (GCSI) continues to increase, indicating the potential for grassland overload and irreversible degradation. There is an urgent need to improve the carrying capacity of grasslands to prevent widespread irreversible degradation.

## Suggestion and discussion

### Discussion

While most of the eastern MP experiences grass-livestock imbalance with overloaded conditions, the reasons behind this overload vary across different regions. In Inner Mongolia, Tongliao and Chifeng have fertile soils and relatively high grassland carrying capacity. However, excessive livestock numbers lead to severe grassland overload in these regions. In contrast, areas like Central Province in MNG experience yearly degradation in grassland productivity, coupled with increased livestock numbers, exacerbating the overload from overloaded to severely overloaded conditions. Conversely, regions such as Bayan-Ölgii in MNG and Ordos City in IMNG suffer from relatively infertile land, resulting in lower grassland carrying capacity and persistent overload throughout the year. Liao et al., using the four-quadrant model, evaluated the overgrazing situation and found that the Hulunbuir and Xilingol League regions require additional livestock numbers, while areas such as Chifeng and Ordos suffer from severe grassland overload. Huang et al., through an analysis of grassland vegetation coverage and forage supply, examined the carrying pressure of livestock on grasslands in China^41^. The results indicate that the central and eastern regions of IMNG have relatively low livestock carrying pressure, still retaining some carrying capacity, the western region was under light carrying pressure in 2015, but with the passage of time, the livestock pressure has increased. Meanwhile, the southern grasslands have already reached a moderate overgrazing state. This is consistent with the results of our study. To address the varying degrees of grassland overload in different regions, it is crucial to thoroughly consider their primary influencing factors and adjust grazing activities accordingly to achieve sustainable utilization of grasslands.

### Suggestions on the development of animal husbandry on MP

As a significant livestock base in Asia and even globally, the MP is a crucial component of the world’s grasslands. Based on the analysis of grassland balance in the MP region, along with spatiotemporal analysis of grassland productivity, the following development recommendations are proposed to address different grass-livestock balance conditions in the MP:

1. Severe Overload Areas: The regions experiencing severe overgrazing are primarily located in the western provinces of MNG and three cities (Chifeng, Tongliao, Hinggan League) in eastern IMNG. Although these areas do not have the best grassland growth conditions, they bear significant responsibilities for livestock farming and have been in a state of severe overgrazing for an extended period. Comprehensive and multifaceted remediation measures are urgently needed to improve the ecological environment and ensure the sustainable utilization of the grassland ecosystem. For instance, the ONE BILLION TREE NATIONAL MOVEMENT, implemented in 2021, and Inner Mongolia’s policy of converting farmland back to forests can significantly improve the ecological environment and enhance the grassland ecosystem. Additionally, it is necessary to adjust regional grazing practices to reduce the pressure on grassland carrying capacity, addressing the overgrazing issue at its root. Measures such as implementing a rotational grazing system, adjusting the layout of livestock production, and modifying grazing practices can prevent overgrazing and overexploitation of resources, thereby reducing the occurrence of grassland overload.
2. Overloaded Areas: The overgrazing areas are primarily located in the central provinces of MNG and Ulanqab City in central IMNG. Grassland productivity in these regions fluctuates between standard and overloaded states depending on the annual grass yield. Therefore, it is essential to strengthen grassland production monitoring and early warning systems. For example, establishing a grassland ecological environment monitoring network that covers significant types and critical areas of grasslands, including monitoring points for indicators such as grassland vegetation cover, grass yield, and the degree of grassland degradation, can enable comprehensive monitoring of grassland growth conditions. In 2023, the first national positioning observation and research station for the grassland ecosystem was established in Hinggan League, Inner Mongolia. The construction of such observation stations should be promoted across the entire MP to provide technical support for effectively preventing and managing grassland overloading. In addition to timely monitoring of grassland growth, measures such as transferring part of the livestock industry to areas with regular or light carrying capacity or introducing livestock feed from lightly loaded areas should be implemented to reduce the grassland carrying pressure within the region.
3. Normal and Lightly Loaded Areas: To maintain the condition of grasslands in normal and lightly loaded areas, monitoring in these regions should be strengthened. These areas are mainly concentrated in the border regions between IMNG and MNG and are also highly prone to grassland fires. Grassland fires pose significant obstacles to the region’s grassland production and livestock industry development. Comprehensive management measures are needed to address the risks and challenges of fire occurrences in this area^42–44^. For example, utilizing remote sensing technology, meteorological monitoring, and fire risk assessment can help promptly assess the likelihood and severity of wildfires. Strengthening international cooperation and information exchange to address transboundary fire risks collectively is also crucial. The signing of the “Agreement between the Government of the People’s Republic of China and the Government of MNG on Joint Prevention and Control of Forest and Grassland Fires in Border Areas” in 2023 has enhanced fire prevention measures in the border regions.

## Conclusion

This study uses multi-source remote sensing data and livestock industry statistics to evaluate the aboveground biomass of grasslands and the grass-livestock balance on the MP from 2018 to 2022. The study concludes the following:

1. The aboveground biomass of grasslands on the MP exhibits significant spatial heterogeneity, showing a gradual increase from southwest to northeast.
2. The total livestock population and standard livestock units on the MP have shown a stable growth trend. Additionally, Mongolia’s livestock population growth rate significantly exceeds that of the Inner Mongolia region.
3. The average carrying capacity of grasslands on the MP reaches 1.8 livestock units per hectare (SU/ha). Inner Mongolia has a higher number of high-quality grasslands, resulting in a significantly better average carrying capacity compared to grasslands in MNG.
4. From 2018 to 2022, the grass-livestock balance on the MP significantly deteriorated, with only a few areas maintaining grasslands in a non-overloaded state.

Based on these conclusions, this paper provides targeted recommendations for grassland management in different carrying capacity regions of the MP, aiming to provide a scientific basis for the sustainable development of the livestock industry in the region.

## Materials and methods

### Study area

**Fig. 5:**
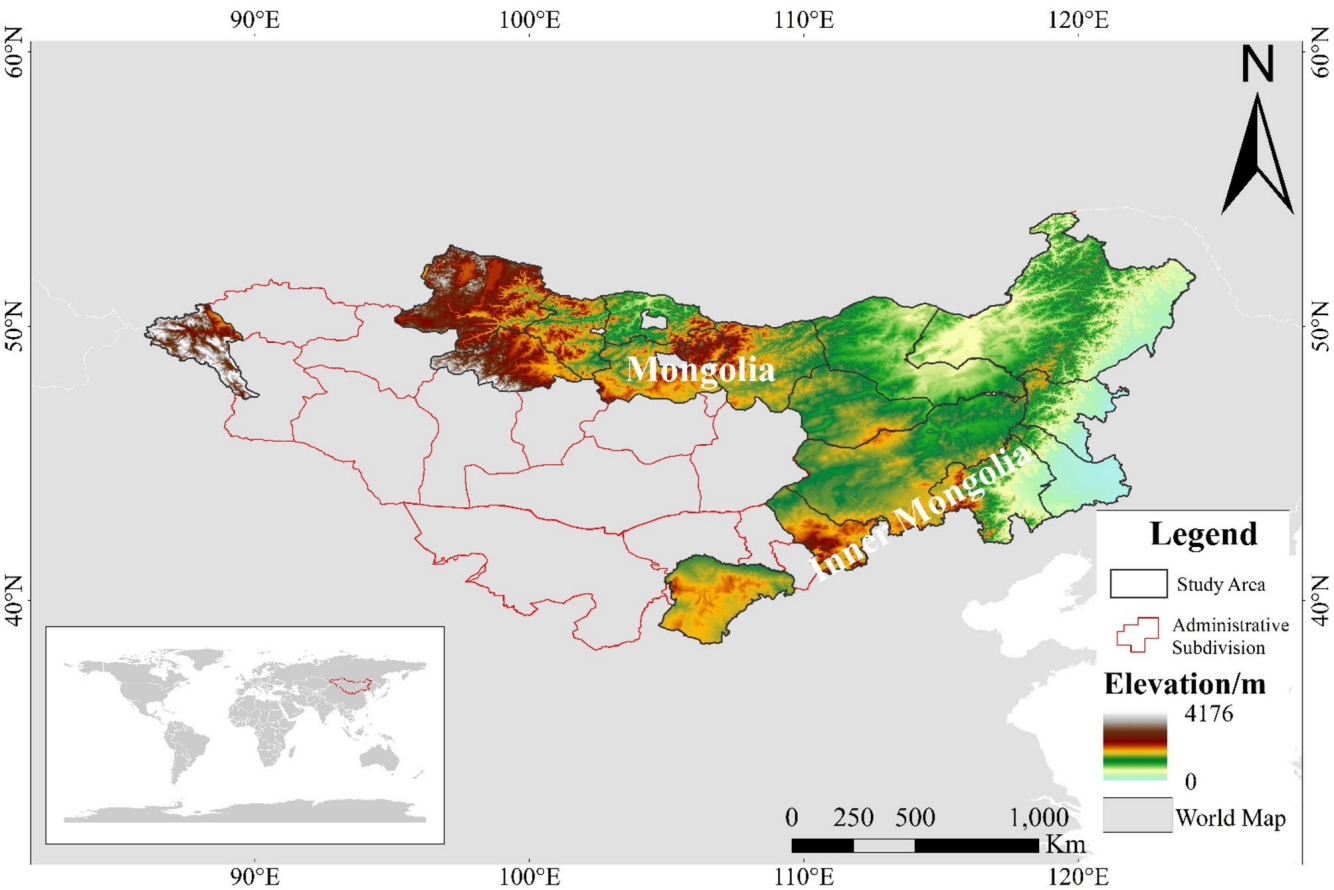
Study area.

The MP is in the central-northern part of Asia, bordered by the Greater Khingan Range to the east and the Altai Mountains to the west, as shown in (Fig. 5). To the north lie the Sayan Mountains and the Yablonoi Mountains, while to the south are the Yinshan Mountains. It spans approximately 37–53°N latitude and 84–126°E longitude, covering most of MNG and the IMNG Autonomous Region of China (hereafter referred to as Inner Mongolia)^45,46^. The MP has a relatively high average elevation, with most areas exceeding 1,000 m. Its highest point is located at the intersection of the Altai Mountains and the Hangai Mountains, with peaks reaching over 4,000 m above sea level. The overall terrain gradually descends from west to east. The MP experiences a temperate continental monsoon climate characterized by significant seasonal variations. Winters are cold and prolonged, with temperatures dropping below − 30 °C, leading to frequent frost and snow events. Summers are hot, with considerable temperature differences between day and night, with daytime temperatures soaring up to 40 °C. Spring and autumn are relatively short and prone to sudden climatic events such as dust storms. The study area encompasses grassland-rich provinces on the MP, including eight provinces in MNG: Bayan-Ölgii, Bulgan, Dornod, Khentii, Khovsgol, Selenge, Sukhbaatar, and Ulaanbaatar. In IMNG, the selected areas include seven cities: Chifeng, Tongliao, Ordos, Hulunbuir, Ulanqab, Xing’an League, and Xilin Gol League.

### Data sources and preprocessing

#### Remote sensing data

The selected remote sensing data parameters for this study are shown in (Table 2). The NDVI data used in this study are sourced from the MOD13Q1 dataset provided by GEE, with a temporal resolution of 16 days. Using the Maximum Value Composite method, NDVI data for the summer months (June-August) on the MP since 2018 have been generated. This method helps mitigate abrupt data fluctuations caused by atmospheric noise, such as clouds and aerosols. The land surface temperature (LST) data are sourced from the MOD11A2 dataset provided by the Google Earth Engine platform, with a temporal resolution of 8 days. Data processing has generated daily average land surface temperature data for the summer months on the MP, with units in degrees Celsius^47,48^. The precipitation data are sourced from the PERSIANN-CDR dataset provided by NOAA, with spatial and temporal resolutions of 0.25 degrees and one day, respectively. After processing, the total summer precipitation data for the MP have been obtained. This dataset utilizes the PERSIANN algorithm, which leverages GridSat-B1 infrared satellite data to extract total precipitation amounts. It possesses high data accuracy and shows good application prospects in arid regions. The remote sensing data used in the above analysis were from 2018 to 2022, and the Filter function was applied for data selection, with functions such as max and mean used for monthly data synthesis. A model was constructed using the three mentioned datasets and ground-based measurements of grassland above-ground biomass to estimate the grassland AGB on the MP from 2018 to 2022^49^. The grassland productivity on the MP is categorized into five classes: extremely low (< 200 g/m^2^), low (200–250 g/m^2^), moderate (250–300 g/m^2^), high (300–350 g/m^2^), and extremely high (> 350 g/m^2^).

**Table 2:** Remote sensing data.

Abbreviation	Resolution	Temporal resolution	Data source
NDVI	500 m	16d	MOD13Q1
LST	1000 m	8d	MOD11A2
Precipitation	0.25°	1d	PERSIANN-CDR

#### Land cover data

The land cover data utilized in this study is the consequence of training based on the random forest algorithm by means of Landsat8 image data. The training samples were established firstly. The land cover data of environmental systems research institute (ESRI), European space agency (ESA) and dynamic world were superimposed to obtain the region with the same land cover classification; Then 2500 training sample points were extracted in this region, and the training sample points were uploaded to GEE; Landsat8 was selected as image data in GEE consequently, and its 6 visible bands, NDVI, NDWI, DEM and slope were taken as classification features. Finally, the random forest model was used to generate land cover classification data in 2020.

**Fig. 6:**
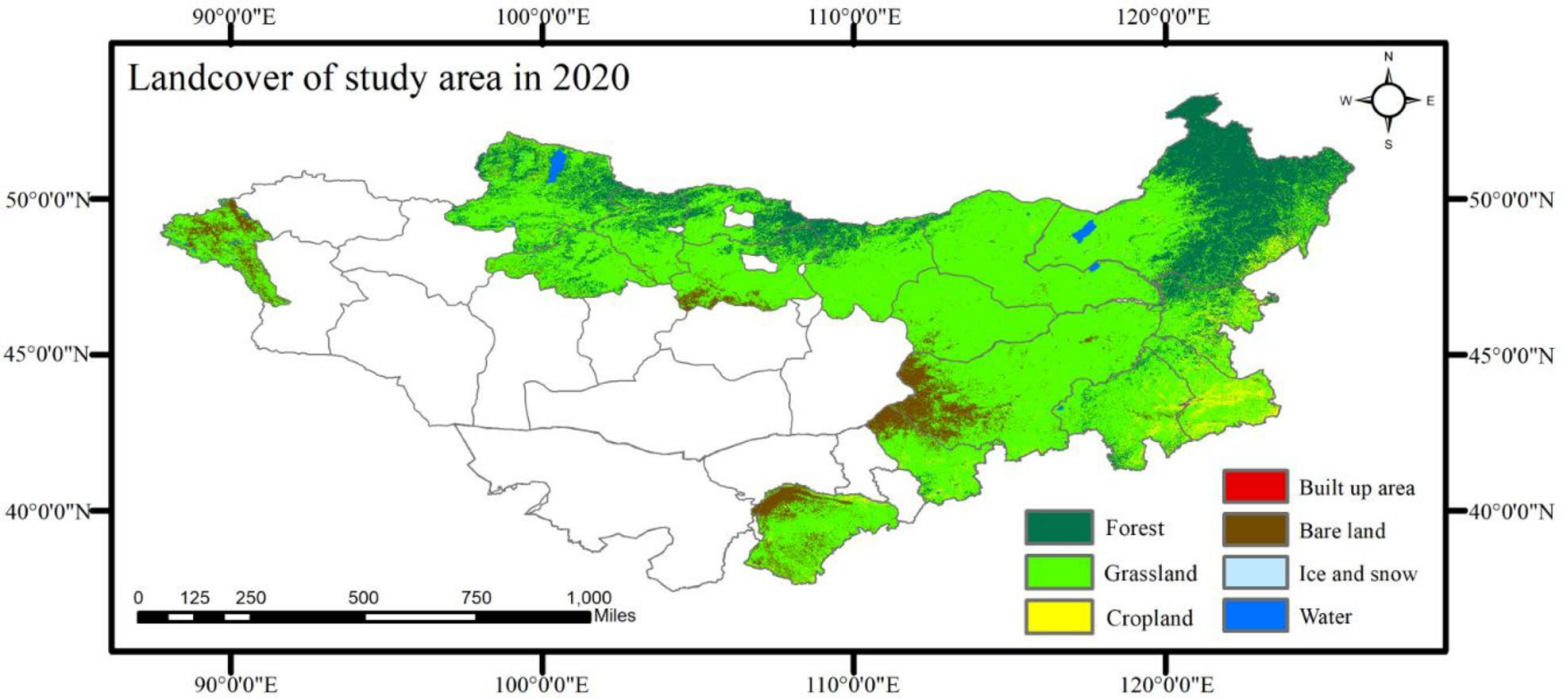
The land cover data for the Mongolian Plateau in 2020.

As a result, 1,500 validation samples were selected for accuracy assessment based on visual interpretation using Google Earth Pro. The land cover results are shown in (Fig. 6). The overall classification accuracy was 93.88%, with a Kappa coefficient of 0.902. The precision and recall rates for grassland classification were 93.2 and 88.5%, respectively, indicating precise delineation of grassland areas on the MP. Grassland covers 47.09% of the area, bare land covers 34.6%, and forests cover 14.98%, representing the three primary land cover types on the MP.

#### Livestock data

The livestock data for MNG are sourced from the statistical information released by the National Statistical Office of Mongolia (https://1212.mn/mn). In contrast, the IMNG livestock data are sourced from the Inner Mongolia Statistical Yearbook published by the Inner Mongolia Bureau of Statistics (http://tj.nmg.gov.cn/).

#### Calculation of grassland carrying capacity

The carrying capacity of grassland is a critical indicator for evaluating the balance between grass and livestock, and it is crucial for achieving the coordinated development of grassland ecology and animal husbandry^50,51^. Its basic equivalent is the theoretical stocking rate, which represents the number of animal units that can be grazed within a given period. The commonly used method for estimating grassland carrying capacity is based on the grass production within the region, with the specific calculation formula as follows:

1 $$GC{C_i}=\frac{{\sum {AG{B_i}*{A_i}*(1 - FU)} }}{{Int*D*1000}}$$

Where $GC{C_i}$ represents the carrying capacity of grassland in the city i, $AG{B_i}$ denotes the average grass production in that city, and 0.2 is set as the utilization level explaining the loss of forage due to trampling, decomposition, and consumption by other herbivores. $Int$represents the daily grass intake of a standard livestock unit, and *D* represents the number of grazing days, set as 180 days for the overall grazing situation on the MP. When evaluating grassland carrying capacity, it is necessary to convert different types and sizes of herbivores into standardized livestock units (LU). In China, according to the National Agricultural Industry Standard (NY/T 635–2002), these are typically converted into Standardized Sheep Units (SU), with each Standardized Sheep Unit having a daily forage intake of 1.8 kg.

The condition of grassland carrying capacity is an essential indicator for assessing the balance between grass and livestock and evaluates how well the grassland ecosystem meets livestock grazing needs. It is typically calculated by comparing livestock quantity with the theoretical stocking rate. This study uses the grassland carrying state index (GCSI), which evaluates the balance between grass and livestock on the MP from the perspective of grassland resource supply and consumption^52,53^. The calculation formula is as follows:

2 $$GCS{I_i}=\frac{{L{N_i}}}{{GC{C_i}}}$$

Where $GCS{I_i}$ represents the grassland carrying state index for city *i*, and $L{N_i}$ denotes the actual number of standard livestock units in that city. According to livestock statistics, the livestock industry in MNG primarily consists of five types of animals: sheep, goats, horses, cattle, and camels, while Inner Mongolia mainly focuses on six types of livestock: cattle, horses, sheep, donkeys, mules, and camels. According to the Agricultural Industry Standard of the People’s Republic of China (NY/T 635–2002), the conversion of regional standard livestock units is based on the end-of-year livestock inventory in the region. One cow is equivalent to 5 standard livestock units, one horse equals 6 standard livestock units, one donkey equals 3 standard livestock units, one mule equals 5 standard livestock units, one camel equals 7 standard livestock units, one sheep equals 1 standard unit, and one goat equals 0.9 standard units. IMNG does not distinguish between sheep and goats, so one sheep is converted to 0.95 standard units.

According to the GCSI, the grass-livestock balance status of various provinces and cities on the MP is categorized into four levels to differentiate among regions. The range of GCSI corresponding to each level is shown in the Table 3 below.

**Table 3:** Classification of grassland carrying state.

GCSI	< 0.8	0.8–1.0	1.0–1.3	> 1.3
Loaded state	Light load	Normal	Overload	Severe overload

## Data Availability

The data that supports the findings of this study are available from the corresponding authors upon request.
